# The Effects of UV-LED Technology on the Quality of Ready-to-Eat Pomegranates: Epigenetic Indicators and Metabolomic Analysis

**DOI:** 10.3390/foods14132192

**Published:** 2025-06-23

**Authors:** Aihemaitijiang Aihaiti, Yuanpeng Li, Xinmeng Huang, Yuting Yang, Ailikemu Mulati, Jiayi Wang

**Affiliations:** College of Life Science and Technology, Xinjiang University, Urumqi 830046, China; aa2386935884@xju.edu.cn (A.A.);

**Keywords:** produce processing, metabolites, disinfection, preservation

## Abstract

Pomegranates are rich in nutrients and classified among ready-to-eat fruits and vegetables. Although this ready-to-eat produce offers convenience, it presents risks associated with pathogenic microorganisms, highlighting the need for pre-sale disinfection. Ultraviolet light-emitting diodes (UV-LEDs) constitute an innovative non-thermal processing technology for food products, offering reduced heat generation and lower energy consumption compared to traditional ultraviolet (UV) irradiation methods. This study analyzed the effects of UV-LED technology on pomegranate seed quality over 0 to 5 days of storage. The results demonstrated significant increases in anthocyanins, polyphenols, ascorbic acid, and the antioxidant capacity in pomegranate following treatment, peaking on day 3. In contrast, the control group showed declining trends. After treatment, the aerobic mesophilic counts and counts of mold and yeast levels during storage measured between 2.73–3.23 log CFU/g and 2.56–3.29 log CFU/g, respectively, significantly lower than the control group. Non-targeted metabolomic analysis showed that UV-LED treatment prompted modifications in the biosynthetic pathways of flavonoids, flavonols, and anthocyanins. The expression of peonidin-3-O-rutinoside chloride increased by 46.46-fold within the anthocyanin biosynthesis pathway. In conclusion, UV-LED treatment represents a potential approach to the disinfection of ready-to-eat fruits and vegetables.

## 1. Introduction

The pomegranate fruit is categorized among ready-to-eat fruits and vegetables, and is characterized by a distinctive and complex flavor profile that integrates sweetness, acidity, and subtle floral notes, resulting in a rich texture [1]. Pomegranate is rich in polyphenols, gallic acid tannins, rosin tannins, flavonols, anthocyanins, vitamins, and various other essential organic nutrients [2]. These compounds demonstrate antioxidant properties, modulate lipid levels, and exhibit antitumor, anti-inflammatory, and antimutagenic effects [3]. Ready-to-eat fruits and vegetables, commonly known as fresh-cut produce, provide benefits regarding convenience and variety. These characteristics appeal to numerous contemporary consumers, particularly those with limited temporal resources. The progressive substitution of conventional processed foods with minimally processed, fresh, and nutritious alternatives is predominantly attributable to heightened consumer awareness of food safety [4]. This heightened consumer awareness has significantly contributed to the rapid expansion of the ready-to-eat fruits and vegetables industry [5]. However, ready-to-eat fruits and vegetables are more susceptible to spoilage due to minimal processing methods, and are easily contaminated by pathogenic microorganisms (*Salmonella*, *Escherichia coli*, etc.) during peeling, cutting, disinfection, color protection, pre-cooling and refrigeration, transportation, and storage, resulting in a decline in nutritional quality and endangering human health [6]. In October 2023, an outbreak of *Salmonella* contamination in fresh-cut onions led to 80 reported cases of illness, 18 hospitalizations, and one fatality across 23 states [7]. The prolonged exposure of ready-to-eat fruits and vegetables to atmospheric environmental conditions may enhance the activity of polyphenol oxidase (PPO) and ethylene. This phenomenon results in various detrimental effects, including the browning of surfaces, the loss of juice and the softening of tissues, which ultimately diminish the storage longevity of fruits and vegetables [8].

UV irradiation is conventionally employed as a method for sterilizing surfaces, effectively achieving this without leaving any residues. UV technology is typically categorized into three types: UV-A (320–400 nm), UV-B (280–320 nm), and UV-C (200–280 nm) [9]. While excessive exposure to UV-C radiation can result in cellular and DNA damage, moderate exposure to UV-C may facilitate the synthesis of bioactive compounds in ready-to-eat fruits and vegetables. Furthermore, it has the potential to reduce respiration rates, thereby decelerating the aging process [10]. Moreover, exposure to UV radiation results in the formation of cyclobutene–pyrimidine dimers and pyrimidine–pyrimidine–pyrimidinone photoproducts in microbial cell DNA. These photoproducts exhibit both cytotoxic and mutagenic properties, possessing the potential to disrupt intracellular DNA replication and transcription, which may lead to microbial cell death [7]. UV-C irradiation facilitates the synthesis of stress-related compounds in fruits and vegetables, including chitinase, β-1,3-glucanase, phenylalanine ammonia-lyase (PAL), and other defense enzymes [10]. Adhikari [11] demonstrated that a 10 s exposure of fresh apples to UV-C irradiation resulted in a reduction in *E. coli O157:H7* and *L. monocytogenes* by 2.1 and 1.6 log CFU/g, respectively. Syamaladevi [12] reported that subjecting fresh pears to UV-C irradiation for a period of 4 min resulted in a reduction in *E. coli O157:H7* by 3.7 log CFU/g. Manzocco [13] found that the application of UV-C treatment to fresh-cut apples resulted in a reduction in the total viable count by 2 log CFU/g in the treated group compared to the blank control group. A study by Martínez-Hernández [14] demonstrated that the application of UV-C treatment effectively inactivated *Streptococcus enteritidis* and *L. monocytogenes* in fresh-cut broccoli.

UV-LED technology represents an emerging category within non-thermal food-processing technologies. It is characterized by reduced heat generation, lower energy consumption, an extended operational lifespan, and enhanced stability in irradiation power [15]. Due to its advantages, UV-LED technology has been implemented for the sterilization of produce such as blueberries, carrots, and spinach [16]. UV-LED is effective in achieving disinfection and sterilization. The physiological mechanism underlying this effect may involve the induction of DNA mutations in pathogenic microorganisms located on the surfaces of fruits and vegetables, as well as the disruption of microbial cell membranes, ultimately resulting in microbial death [17].

In this study, we employed Xinjiang pomegranate as the raw material to investigate and compare the differences in epigenetic indicators between the UV-LED-treated group and the blank control group. Furthermore, we conducted an analysis of the differences in pomegranate metabolites between the two groups utilizing non-targeted metabolomics.

## 2. Materials and Methods

### 2.1. Chemicals, Reagents and Instruments

Xinjiang pomegranates (*Punica granatum* L.) were purchased from the Urumqi Jiuding Wholesale Market (December 2024); these contained a 78.6% moisture content. The experiments were conducted on the same day the pomegranates were purchased. For the experiments, fruits were selected based on the following criteria: freshness and ripeness, the absence of visible exterior damage, freedom from insect infestation, and a uniform dark red coloration without any abnormal hues. LC-MS grade acetonitrile (ACN) and methanol (MeOH) were purchased from Fisher Scientific (Loughborough, UK). Chloroform was obtained from Sinopharm (Shanghai, China). Ultrapure water was generated using a Milli-Q system (Millipore, Bedford, MA, USA). 2-Amino-3-(2-chloro-phenyl)-propionic acid was purchased from Aladdin (Shanghai, China). Formic acid was obtained from TCI (Shanghai, China). Ammonium formate was obtained from Sigma-Aldrich (Shanghai, China). Ultrapure water was generated using a Milli-Q system (Millipore, Bedford, MA, USA).

### 2.2. Sample Preparation

A UV-LED chamber (390 × 300 × 260 mm, Jigu, Zhongshan, China) was equipped with 200 UV-LED bulbs that were uniformly distributed across the top of the chamber. Pomegranates of superior quality were cleaned, peeled, and deseeded, after which they were evenly divided into two groups and placed in trays. One group was subjected to irradiation in an UV-LED chamber for a duration of 10 min. The irradiation intensity was 2.1 ± 0.1 mW/cm^2^, measured using an irradiator (Linshang, Shenzhen, China). The UV-LED wavelength was 255 ± 1 nm [7], presenting a full bandwidth of 13.7 nm at half the maximum value, measured using a spectral irradiance colorimeter (HPS350UV; Hopoolight, Hangzhou, China). The other group functioned as the control and did not undergo any treatment. Each treatment group comprised four biological replicates. The samples were stored in fruit and vegetable boxes (175 × 110 × 75 mm), with approximately 40 g of pomegranate seeds placed in each box. The boxes were sealed with sealing film and stored at 4 °C under refrigeration conditions.

### 2.3. Measurement of Physical and Chemical Indicators

#### 2.3.1. Determination of Polyphenol Content

Two sets of pomegranate seed samples were collected on days 0, 1, 3, and 5 of storage. The pulp was ground in a pulverizing grinder using liquid nitrogen until samples were reduced to a white powder. The powder was introduced into a centrifuge tube, and the volume was capacitanced to 5 mL using an 80% methanol solution. The mixture was sonicated in a water bath for 20 min, followed by centrifugation at 4 °C and 11,000 rpm for 10 min; then, the supernatant was extracted. A volume of 50 μL of the supernatant and 250 μL of Folin reagent was added to 3 mL of distilled water for a duration of 6 min. Subsequently, 750 μL of a 20% sodium carbonate solution was added. The reaction was conducted at room temperature and was shielded from light for 45 min. The absorbance was measured at 765 nm, and the results were expressed as micrograms of gallic acid equivalents per milliliter (µg GAE/mL) [18].

#### 2.3.2. Determination of Total Antioxidant Capacity (T-AOC)

A total antioxidant capacity (T-AOC) assay kit (A015-1-2, Nanjing Jiancheng Bioengineering Institute, Nanjing, China) was used to determine the total antioxidant capacity. The results were expressed as mg/kg fresh weight.

#### 2.3.3. Determination of Anthocyanin Content

Two aliquots were prepared: one with a KCl buffer solution (0.025 M, pH = 1.0) and the other with a CH_3_COONa buffer (0.4 M, pH = 4.5). A volume of 0.5 mL of the supernatant obtained from the polyphenol assay was combined with 4.5 mL of buffer solution and allowed to stand at ambient temperature for 20 min, shielded from light. The absorbance of the resulting mixture was measured at wavelengths of 520 nm and 700 nm, respectively [19]. The total anthocyanin content was subsequently calculated using the following equation (the results were expressed as mg/g fresh weight.):

(1) $$\frac{A\times 449.2\times 10\times 1000}{269,000\times 1}\;A\;(\mathrm{absorbance})=(A520\;\mathrm{nm}–A700\;\mathrm{nm}\;\mathrm{pH}=1.0)-(A520\;\mathrm{nm}–A700\;\mathrm{nm}\;\mathrm{pH}=4.5)$$

#### 2.3.4. Determination of Ascorbic Acid Content

An ascorbic acid (ASA) assay kit (A009-1-1, Nanjing Jiancheng Bioengineering Institute, Nanjing, China) was used to determine the ascorbic acid content. The results were expressed as mg/g fresh weight.

#### 2.3.5. Determination of Microbiological Indicators

Fifteen grams of pomegranate seeds was combined with 85 mL of sterile saline and subjected to centrifugation at 37 °C and 260 rpm for 2 min. Subsequently, 20 mL of Bengal red, for the analysis of mold yeast, and nutrient agar, for the assessment of the total number of colonies, were dispensed into a sterile Petri dish and thoroughly mixed. Then, 1 mL of bacterial suspension was 10-fold serially diluted in 9 mL of sterile water. Then, 100 μL of the mixed-samples suspension was plated onto the above medium according to the spread plate method. The total number of colonies was analyzed following a 2-day incubation period at 37 °C, while mold yeasts were assessed after a 5-day incubation at 28 °C. The results were expressed as logarithmic CFU/g.

### 2.4. Metabolomic Analysis

#### 2.4.1. Liquid Chromatography Conditions

A sample of pomegranate seeds was taken on the 3rd day of storage and the pulp was ground in liquid nitrogen and placed in a 2 mL centrifuge tube. Then, 1000 µL of tissue extract [75% (9:1 methanol: chloroform):25% H_2_O] was added, along with steel balls. This was put into the tissue grinder and ground at 50 Hz for 60 s, with the above operation repeated twice. Then, room-temperature ultrasound was performed for 30 min and ice bath ultrasound was performed for 30 min. This was centrifuged for 10 min at 12,000 rpm and 4 °C before all the supernatant was transferred to a new 2 mL centrifuge tube and concentrated and dried. Then, 200 µL of 50% acetonitrile solution was prepared and added to 2-chloro-l-phenylalanine (4 ppm) to re-dissolve the sample, after which the supernatant was filtered by a 0.22 μm membrane and transferred into the detection bottle for LC-MS detection [20].

The LC analysis was performed on a Vanquish UHPLC System (Thermo Fisher Scientific, Waltham, MA, USA). Chromatography was carried out with an ACQUITY UPLC ^®^ HSS T3 (2.1 × 100 mm, 1.8 µm) (Waters, Milford, MA, USA). The column was maintained at 40 °C. The flow rate and injection volume were set at 0.3 mL/min and 2 μL, respectively. For LC-ESI (+)-MS analysis, the mobile phases consisted of (B1) 0.1% formic acid in acetonitrile (*v*/*v*) and (A1) 0.1% formic acid in water (*v*/*v*). Separation was conducted under the following gradient: 0~1 min, 8% B1; 1~8 min, 8~98% B1; 8~10 min, 98% B1; 10~10.1 min, 98~8% B1; 10.1~12 min, 8% B1. For LC-ESI (−)-MS analysis, the analysis was carried out with (B2) acetonitrile and (A2) ammonium formate (5 mM). Separation was conducted under the following gradient: 0~1 min, 8% B2; 1~8 min, 8~98% B2; 8~10 min, 98% B2; 10~10.1 min, 98~8% B; 10~12 min, 8% B2 [21].

#### 2.4.2. Mass Spectrum Analysis

The mass spectrometric detection of metabolites was performed on Orbitrap Exploris 120 (Thermo Fisher Scientific, Waltham, MA, USA) with an ESI ion source. Simultaneous MS1 and MS/MS (Full MS-ddMS2 mode, data-dependent MS/MS) acquisition was used. The parameters were as follows: sheath gas pressure, 40 arb; aux gas flow, 10 arb; spray voltage, 3.50 kV and −2.50 kV for ESI (+) and ESI (−), respectively; capillary temperature, 325 °C; MS1 range, 100–1000 *m*/*z*; MS1 resolving power, 60,000 FWHM; number of data-dependent scans per cycle, 4; MS/ MS resolving power, 15,000 FWHM; normalized collision energy, 30%; dynamic exclusion time, automatic [22].

#### 2.4.3. Data Preprocessing

The raw data were firstly converted to mzXML format by MSConvert in the ProteoWizard software package (v3.0.8789) [23] and processed using R XCMS (v3.12.0) for feature detection [24], retention time correction and alignment. The key parameter settings were as follows: ppm = 15, peakwidth = c (5, 30), mzdiff = 0.01, method = cent Wave. The batch effect was then eliminated by correcting the data based on QC samples. Metabolites with RSD > 30% in QC samples were filtered and then used for subsequent data analysis.

The metabolites were identified using accuracy mass and MS/MS data, which were matched with HMDB (http://www.hmdb.ca (accessed on 20 October 2024) [25], massbank (http://www.massbank.jp/ (accessed on 25 October 2024) [26], KEGG (https://www.genome.jp/kegg/ (accessed on 28 October 2024) [27], LipidMaps (http://www.lipidmaps.org (accessed on 5 November 2024) [28], and mzcloud (https://www.mzcloud.org (accessed on 18 November 2024) [29]. The molecular weight of metabolites was determined according to the *m*/*z* (mass-to-charge ratio) of parent ions in MS data. The molecular formula was predicted by ppm (parts per million) and adduction, and then matched with the database to perform the MS identification of metabolites. At the same time, the MS/MS data from a quantitative table of MS/MS data were matched with the fragment ions and other information of each metabolite in the database, so as to realize the MS/MS identification of metabolites.

Two different multivariate statistical analysis models, unsupervised and supervised, were applied to discriminate the groups (PCA; PLS-DA; OPLS-DA) by R ropls package [30]. The statistical significance of the *p* value was obtained via a statistical test between groups. Finally, combined with the *p* value, VIP (OPLS-DA variable projection importance) and FC (multiple of difference between groups) were used to screen biomarker metabolites. By default, when *p* value < 0.05 and VIP value > 1, the metabolite was considered to have significant differential expression.

#### 2.4.4. Pathway Analysis

Differential metabolites were analyzed using MetaboAnalyst [31], which combines the results of powerful pathway enrichment analysis with pathway topology analysis. The metabolites identified in metabolomics were then mapped to the KEGG pathway to facilitate the biological interpretation of higher-level systemic functions. The visualization of the metabolites and their associated pathways was accomplished using the KEGG Mapper tool.

### 2.5. Statistical Analysis

Three independent biological replicates were conducted for each experimental set, except for metabolome data. Statistical analyses were performed using SPSS 20.0 software (IBM, New York, NY, USA), employing one-way analysis of variance (ANOVA) and paired-samples *t*-tests to determine significant differences. A threshold of *p* < 0.05 was established for statistical significance. All data are presented as mean ± standard deviation.

## 3. Results

### 3.1. Identification and Analysis of Epigenetic Indicators

#### 3.1.1. The Impact of UV-LED Technology on Nutrient Composition of Ready-to-Eat Pomegranate

**Figure 1:**
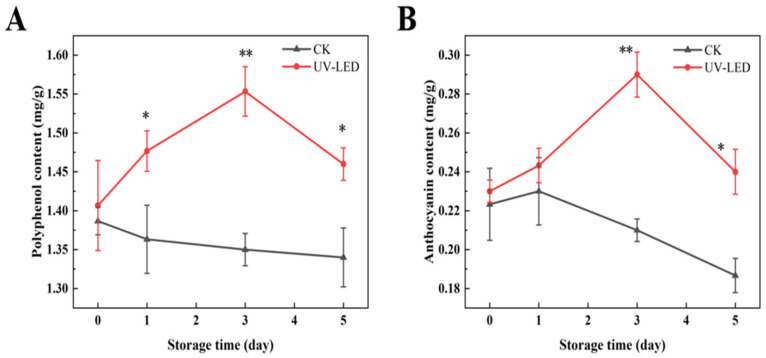
Contents evolution of (**A**) polyphenols and (**B**) anthocyanins achieved by UV-LED on different storage days. The contents evolution indicates the difference between the control and treatment groups. Different asterisks within the same group indicate significant differences (** *p* < 0.01; * *p* < 0.05). US, ultrasound; UV-LED, ultraviolet-light emitting diodes.

Pomegranate contains anthocyanins and polyphenols as its primary bioactive compounds, both of which exhibit antioxidant properties and have the potential to prevent chronic diseases, including cardiovascular disease [32] and diabetes [33]. The concentrations of polyphenol and anthocyanin in the two groups of pomegranate samples exhibited a significant difference, as depicted in Figure 1A, B. During the storage period of 0 to 5 days, the polyphenol and anthocyanin contents in the control group of pomegranates exhibited a declining trend. Conversely, the group exposed to UV-LED exhibited an initial increase followed by a decrease, with the peak observed on the third day. On day 3, the concentrations of anthocyanin and polyphenol in the treated group increased by 0.06 and 0.15 mg/g, respectively, in comparison to the control group. The mechanism is likely attributed to the observation that UV-LED treatment enhances the activity of PAL, thereby promoting the degradation of phenylalanine. This process promotes the conversion of phenylalanine into coumaric acid, which is subsequently converted into a series of polyphenols through the action of phenylketone synthetase and hydroxycoumarate benzyltransferase [34]. The biosynthetic pathway of flavonoids is intricately associated with the synthesis of phenolic acids, with phenylalanine serving as the initial substrate. Furthermore, exposure to UV-LED has been shown to enhance the activities of 4-coumarate-CoA ligase and chalcone synthase (CHS), thereby promoting the production of polyphenols [34]. However, the prolonged storage of pomegranate seeds results in a reduction in the activities of enzymes, such as PAL, phenylacetone synthase, and CHS, which consequently results in a decrease in the levels of polyphenols and anthocyanins [35].

**Figure 2:**
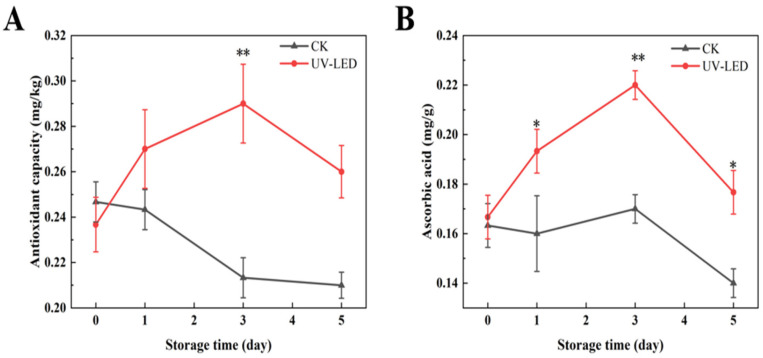
Contents evolution of (**A**) antioxidant capacity and (**B**) ascorbic acid achieved by UV-LED on different storage days. The contents evolution indicates the difference between the control and treatment groups. Different asterisks within the same group indicate significant differences (** *p* < 0.01; * *p* < 0.05). US, ultrasound; UV-LED, ultraviolet-light emitting diodes.

The antioxidant capacity has been demonstrated to positively correlate with the concentrations of anthocyanins and polyphenols [32]. In the present study, the antioxidant capacity reached its maximum on day 3, measuring 0.29 mg/kg, as depicted in Figure 2A. Furthermore, the data indicate that ascorbic acid also exhibits antioxidant efficacy [36], as depicted in Figure 2B, with its peak observed on day 3 at 0.22 mg/g. H_2_O_2_ is a significant ROS generated during the ripening process of fruits and vegetables, which is closely linked to their shelf life [37]. In fruits and vegetables, H_2_O_2_ is generated as a consequence of mitochondrial preoxidation, which plays a role in the softening of these products [38], the production of free sugars [39], and the accumulation of antioxidants [40]. In fact, ascorbic acid effectively inhibits the production of H_2_O_2_, thereby facilitating the maintenance of stable cell morphology [41].

#### 3.1.2. The Impact of UV-LED Technology on Microbial Indicators of Ready-to-Eat Pomegranate

**Figure 3:**
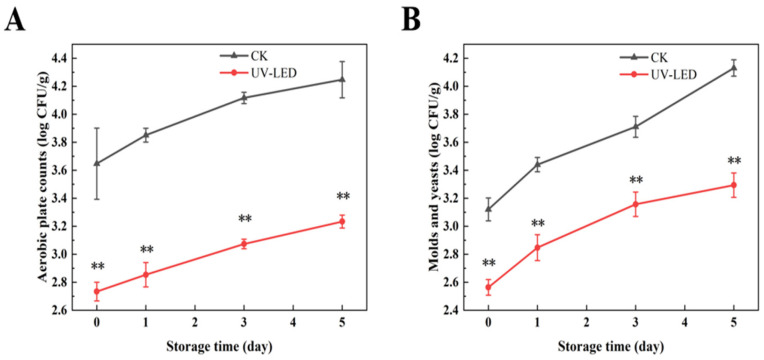
Counts evolution of (**A**) aerobic plate and (**B**) molds and yeasts achieved by UV-LED on different storage days. The counts evolution indicates the difference between the control and treatment groups. Different asterisks within the same group indicate significant differences (** *p* < 0.01). US, ultrasound; UV-LED, ultraviolet-light emitting diodes.

As depicted in Figure 3A,B, the aerobic plate counts and mold yeast counts in the UV-LED-treated group of pomegranates were significantly lower than those in the blank control group. On the third day following UV-LED treatment, the aerobic plate counts and mold yeast counts were recorded at 3.07 and 3.16 log CFU/g, respectively. This indicates a reduction of 1.05 and 0.55 log CFU/g compared to the blank control group. This may be attributed to the UV-LED treatment’s inhibition of microbial DNA replication and transcription [15], as well as the disruption of the cell membrane structure, which induces oxidative stress within the cells, thereby contributing to microbial death [42]. The microbial counts across all treatment groups remained below 5 log CFU/g for a duration of 5 days of storage, without surpassing the established safety threshold [43].

### 3.2. PCA and OPLS-DA Analysis

**Figure 4:**
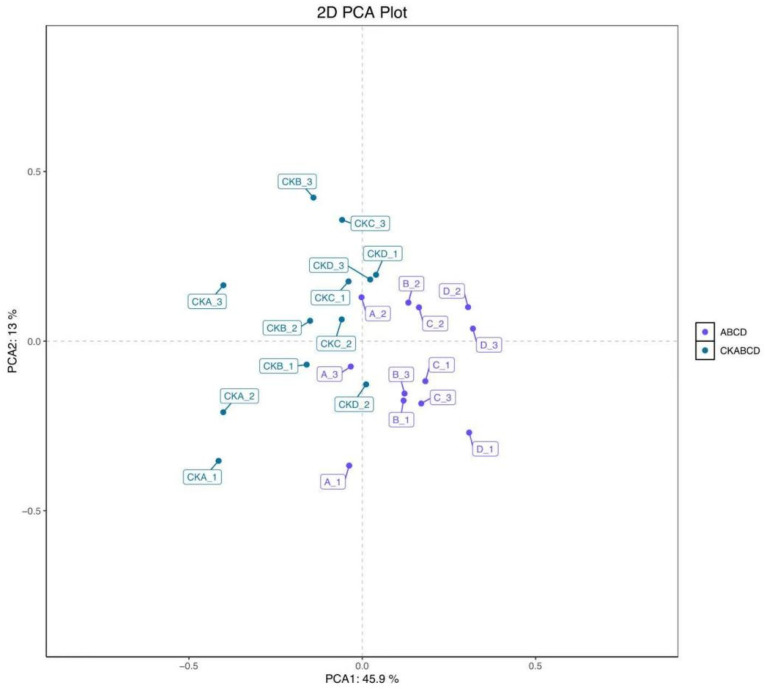
PCA score plots of metabolomic features in both the blank control and UV-LED groups.

The maximum concentrations of anthocyanin, polyphenol, ascorbic acid, and antioxidant capacity in pomegranate were observed on day 3 of storage following UV-LED treatment. Consequently, selecting a pomegranate on day 3 of storage as the sample for the metabolomic assay was most appropriate. Principal Component Analysis (PCA) is an unsupervised chemometric method employed for exploratory data analysis, which facilitates the identification of the distribution of control and treated samples and elucidates differences between samples. PCA and Orthogonal Partial Least Squares Discriminant Analysis (OPLS-DA) were employed to categorize the pomegranate samples into groups treated with UV-LED (A, B, C, and D) and blank control groups (CKA, CKB, CKC, and CKD). Each group underwent four biological replicates. The results of the PCA are presented in Figure 4, with highly significant differences between groups and high clustering within groups [44]. This suggests substantial variability between the metabolites of the blank control group and those of the UV-LED-treated group, thereby confirming the validity of the model predictions.

**Figure 5:**
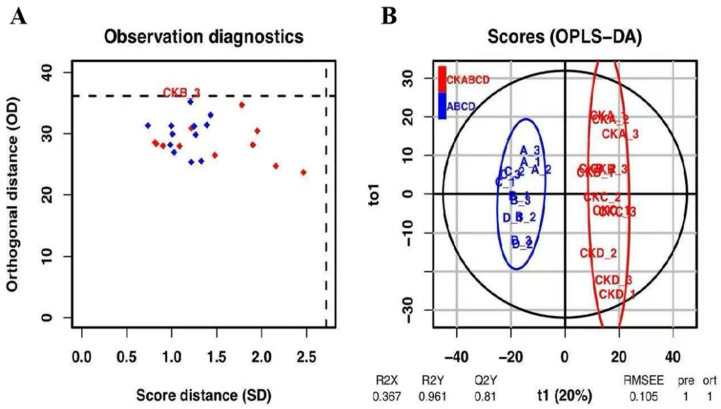
Diagnostic diagram of the OPLS-DA model observations in the blank control group and the UV-LED group, red diamonds represent the control group; blue diamonds represent the treatment group; black dash lines represent the critical value of the model (**A**), and the scoring diagram (**B**).

**Figure 6:**
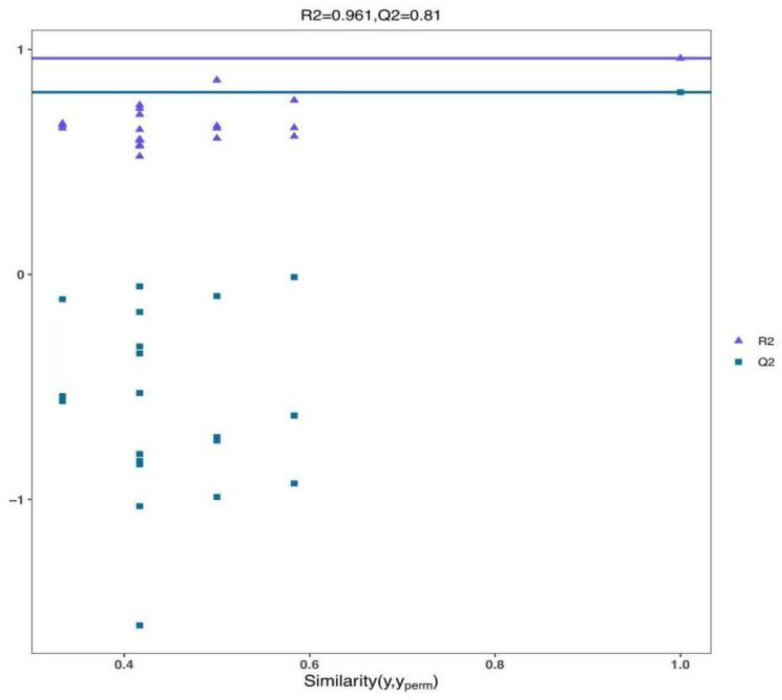
Confidence parameter plots of the OPLS-DA model for both the blank control and UV-LED groups.

OPLS-DA modeled the resultant data for prediction, as depicted in Figure 5 and Figure 6. Based on the diagnostic analysis presented in Figure 5A, it can be inferred that the consistent length of the distances of each sample in both the projection plane and the orthogonal projection plane, coupled with the absence of anomalous values, suggests that the model is devoid of anomalous samples, thereby affirming the reliability of the results. In the OPLS-DA score plot (Figure 5B), it is evident that the samples from the UV-LED-treated group are predominantly located in quadrants two and three, whereas the blank control group is primarily situated in quadrants one and four [44]. This distribution indicates a significant variation in metabolites between the two groups, thereby corroborating the findings of the PCA analysis.

As depicted in Figure 6, the judgment model’s credibility parameters, pR2Y (the degree of model interpretability) and pQ2 (the degree of model predictability), were 0.961 and 0.81, respectively. Both values exceed 0.8, indicating that the model exhibits high credibility and demonstrates a good fit. Therefore, it is deemed suitable for the subsequent identification and screening of differential metabolites.

### 3.3. Enrichment Analysis of KEGG Pathway

**Figure 7:**
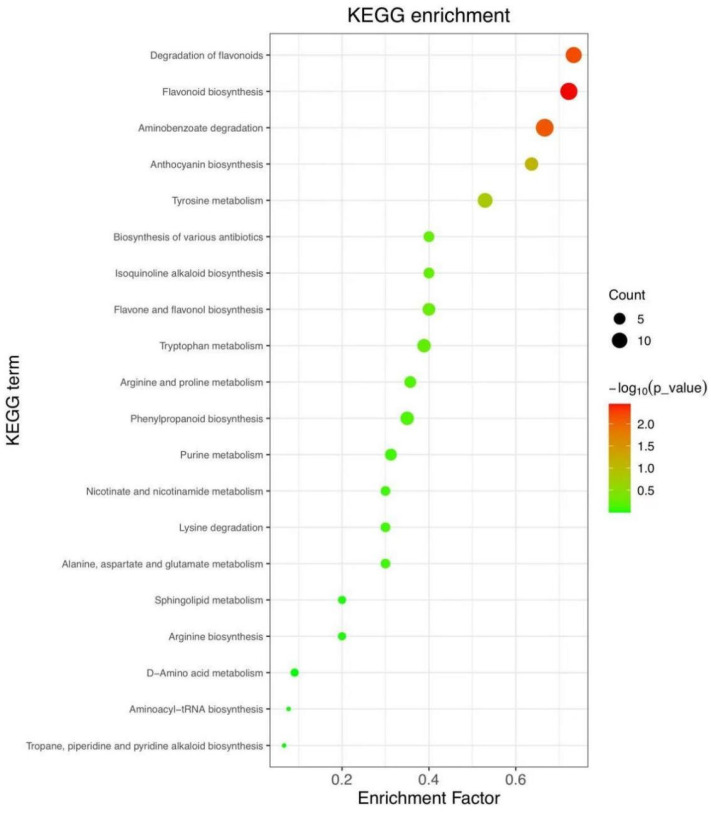
Enrichment analysis of KEGG pathway: Mapping the Top 20 Significant Metabolic Pathways.

We conducted an analysis and comparison of the variations in pomegranate metabolites between the blank control and UV-LED-treated groups utilizing KEGG-enriched pathway analysis. This methodology aimed to characterize the impact of UV-LED on metabolite alterations in pomegranate and to elucidate the underlying physiological mechanisms, thereby identifying the signaling pathways associated with these metabolite changes. As depicted in Figure 7, the anthocyanin synthesis pathway exhibited a significant variation (*p* < 0.01). The variation in anthocyanin accumulation in pomegranates, which imparts distinct colors to the pomegranate pericarp such as red, reddish-purple, pink, and violet, is attributed to secondary metabolites synthesized via the phenylalanine pathway [45]. Anthocyanins possess a molecular structure characterized by the covalent bonding of glycogens and glycosylated molecules through glycosidic linkages. When the glycosidic hydroxyl group undergoes further modification with aromatic or aliphatic organic acids, it results in the formation of structurally stable acylated anthocyanin derivatives [46]. Anthocyanins are water-soluble pigments that play significant functional roles in maintaining the quality and vibrant color of fruits and vegetables. Consequently, anthocyanins can serve as crucial indicators in the study of fruit and vegetable quality.

Anthocyanins, a subclass of flavonoids, have been the subject of extensive research. To date, more than 600 anthocyanins have been identified and analyzed, with cyanidin, delphinidin, malvidin, pelargonidin, peonidin, and petunidin comprising approximately 95% of the total. Floristein derivatives are extensively found in the majority of plants [47]. Anthocyanins play a crucial role in various physiological processes in plants, including photoprotection [48], and antioxidant defense mechanisms, such as free radical scavenging and the modulation of oxidative stress responses. These functions significantly enhance plant tolerance to abiotic stresses, including ultraviolet radiation, cold, and drought [49]. Simultaneously, variations in the pH levels of anthocyanins within plant cell vesicles lead to a variety of colors. For instance, the pigmentation of petals attracts pollinators; the coloration of fruits and vegetables facilitates seed dispersal; and the accumulation of pigments in seed coats contributes to the regulation of seed dormancy. Collectively, these processes enhance the plant’s biological functions and ecological adaptability [50].

Research on anthocyanins has primarily focused on their synthesis within the biosynthetic pathway [51]. The biosynthesis of flavonoids and anthocyanins occurs in three distinct phases: the cleavage of PAL, flavonoid metabolism, and anthocyanin synthesis [52]. These processes are catalyzed by PAL, CHS, chalcone isomerase (CHI), flavonoid 3-hydroxylase (F3H), dihydroflavonoid 4-reductase (DFR), and anthocyanidin synthase (ANS).

Brugliera [53] demonstrated that PAL enzymatically cleaves phenylalanine to yield cinnamic acid, which functions as a precursor in the biosynthesis of anthocyanins. Furthermore, CHS facilitates the synthesis of chalcones, compounds essential for imparting distinctive coloration to fruits and vegetables.

As demonstrated in Table 1, the KEGG-enriched pathway analysis revealed an increase in seven compounds within the anthocyanin metabolic pathway. These compounds were Chrysanthemin, Delphinidin-3,5-O-diglucoside, Cyanidin-3,5-O-diglucoside, Leucodelphinidin 3-glucoside, Peonidin-3-O-rutinoside chloride, Delphinidin-3-caffeoylglucoside, and Peonidine-3-O-glucoside chloride. Chrysanthemin is the primary anthocyanin present in pomegranates, contributing to their distinctive red and purple coloration [54].

Delphinidin-3,5-O-diglucoside is predominantly found in dark purple pomegranates. The diglucosylation imparts resistance to light and mitigates the decomposition of pomegranate-related products, such as pomegranate juice, during processing stages, including juicing and sterilization [55]. Furthermore, it interacts synergistically with ascorbic acid and ellagic acid to augment the antioxidant capacity of pomegranate [56]. It exhibits inhibitory effects on the proliferation of melanoma cells [57] and demonstrates antimicrobial properties, such as preventing the infection of *Staphylococcus aureus* [58]. Cyanidin-3,5-O-diglucoside, a predominant red pigment found in pomegranate seeds, accumulates during the late ripening stage and exhibits antiglycation properties by inhibiting the formation of advanced glycosylation end products (AGEs), which are associated with antidiabetic effects [59]. Furthermore, Tsuda [60] indicated that Cyanidin-3,5-O-diglucoside interacts with adipocytes, facilitating the secretion of lipocalin and leptin, which are significantly associated with anti-obesity effects. Leucodelphinidin 3-glucoside, predominantly found in the pericarp of pomegranates, acts as a precursor in the biosynthesis of anthocyanins. As a defensive precursor, it undergoes rapid oxidation to form delphinidin when exposed to external mechanical damage, such as squeezing or skin rupture, or when subjected to harmful microbial infection, thereby enhancing antimicrobial activity [61]. Peonidin-3-O-rutinoside chloride, the most significantly altered among the seven anthocyanins, is predominantly present in pomegranates with advanced stages of ripeness and demonstrates anti-inflammatory properties. The compound enhances the anti-inflammatory properties of pomegranate by inhibiting the cyclooxygenase-2 (COX-2) pathway and regulates hepatic cell metabolism to mitigate the risk of diabetes [62]. Delphinidin-3-caffeoylglucoside is exclusively found in the pomegranate pericarp, which may be compromised and dissolved during processing [63]. Furthermore, its antioxidant capacity surpasses that of vitamins C and E and it effectively scavenges oxidized free radicals and preserves cellular stability [64]. Peonidin-3-O-glucoside chloride interacts with the chloride ions present in pomegranate, thereby enhancing its molecular stability [65]. Moreover, it contributes to a reduction in cholesterol and blood pressure levels, enhances capillary permeability, and decreases the risk of cardiovascular disease [66].

## 4. Conclusions

In this study, we investigated the impact of UV-LED technology treatment on the metabolites present in ready-to-eat pomegranate samples. We conducted an analysis of the differential changes in polyphenols, anthocyanins, ascorbic acid, antioxidant capacity, and microbial indices, including aerobic plate counts and mold yeast counts, both before and after treatment. The results demonstrated that the levels of physical and chemical indicators increased following the application of UV-LED treatment to pomegranates for 10 min, with these levels reaching their maximum on day 3 of storage. Throughout the storage period, microbial indicators consistently remained lower than those observed in the control group. Furthermore, we conducted an analysis of flavonoid metabolites in both the UV-LED-treated and blank control groups using KEGG-enriched pathway analysis. The findings revealed significant differences in flavonoid metabolites between the two groups. Significantly, Delphinidin derivatives, Cornflower derivatives, and Paeonia lactiflora derivatives demonstrated the most pronounced enhancement.

In practical terms, UV-LED can inactivate enzymes such as peroxidase (POD) and polyphenol oxidase (PPO). Furthermore, they enhance the levels of ascorbic acid, polyphenols, and anthocyanins in fruits and vegetables, thereby extending their shelf life and delaying the maturation process.

Hurdle technology constitutes a notable advancement in the domain of nonthermal food processing. Future research should prioritize the integration of UV-LED with other novel non-thermal food processing technologies, such as the combination of UV-LED with ultrasound (US) or cold plasma (CP). This approach seeks to determine the optimal processing methods and parameters for industrial-scale food sterilization and disinfection, thereby establishing a theoretical foundation for these applications.

## Figures and Tables

**Table 1:** Identification of anthocyanin compounds in pomegranate using LC-MS in both positive and negative ionization modes after KEGG pathway enrichment analysis.

Metabolite	Formula	mz	Content Change
Chrysanthemin	C_21_H_22_O_11_	449.11	1.24
Delphinidin-3,5-O-diglucoside	C_27_H_31_O_17_	627.16	2.64
Cyanidin-3,5-O-diglucoside	C_27_H_31_O_16_	611.16	1.11
Leucodelphinidin 3-glucoside	C_21_H_24_O_13_	489.10	1.48
Peonidin-3-O-rutinoside chloride	C_28_H_33_O_15_	610.18	46.46
Delphinidin-3-caffeoylglucoside	C_30_H_27_O_15_	645.17	1.53
Peonidine-3-O-glucoside chloride	C_22_H_23_O_11_	463.12	1.28

## Data Availability

The original contributions presented in this study are included in the article. Further inquiries can be directed to the corresponding author.
